# Arginylglycylaspartic Acid-Surface-Functionalized Doxorubicin-Loaded Lipid-Core Nanocapsules as a Strategy to Target Alpha(V) Beta(3) Integrin Expressed on Tumor Cells

**DOI:** 10.3390/nano8010002

**Published:** 2017-12-22

**Authors:** Michelli B. Antonow, Camila Franco, Willian Prado, Aline Beckenkamp, Gustavo P. Silveira, Andréia Buffon, Sílvia S. Guterres, Adriana R. Pohlmann

**Affiliations:** 1 Programa de Pós-Graduação em Nanotecnologia Farmacêutica, Faculdade de Farmácia, Universidade Federal do Rio Grande do Sul, Av. Ipiranga, 2752, Porto Alegre 90610-000 RS, Brazil; mbantonow@gmail.com; 2 Programa de Pós-Graduação em Ciências Farmacêuticas, Faculdade de Farmácia, Universidade Federal do Rio Grande do Sul, Av. Ipiranga, 2752, Porto Alegre 90610-000 RS, Brazil; kmillass@yahoo.com.br (C.F.); alinee-b@hotmail.com (A.B.); andreia.buffon@ufrgs.br (A.B.); silvia.guterres@ufrgs.br (S.S.G.); 3 Departamento de Química Orgânica, Instituto de Química, Universidade Federal do Rio Grande do Sul, Av. Bento Gonçalves, 9500, Porto Alegre 91501-970 RS, Brazil; will_ap20@hotmail.com (W.P.); gustavo.silveira@iq.ufrgs.br (G.P.S.)

**Keywords:** lipid-core nanocapsules, cancer, RGD, Doxorubicin, active drug targeting, surface-functionalization

## Abstract

Doxorubicin (Dox) clinical use is limited by dose-related cardiomyopathy, becoming more prevalent with increasing cumulative doses. Previously, we developed Dox-loaded lipid-core nanocapsules (Dox-LNC) and, in this study, we hypothesized that self-assembling and interfacial reactions could be used to obtain arginylglycylaspartic acid (RGD)-surface-functionalized-Dox-LNC, which could target tumoral cells overexpressing αvβ3 integrin. Human breast adenocarcinoma cell line (MCF-7) and human glioblastoma astrocytoma (U87MG) expressing different levels of αvβ3 integrin were studied. RGD-functionalized Dox-LNC were prepared with Dox at 100 and 500 mg·mL^−1^ (RGD-MCMN (Dox100) and RGD-MCMN (Dox500)). Blank formulation (RGD-MCMN) had z-average diameter of 162 ± 6 nm, polydispersity index of 0.11 ± 0.04, zeta potential of +13.2 ± 1.9 mV and (6.2 ± 1.1) × 10^11^ particles mL^−1^, while RGD-MCMN (Dox100) and RGD-MCMN (Dox500) showed respectively 146 ± 20 and 215 ± 25 nm, 0.10 ± 0.01 and 0.09 ± 0.03, +13.8 ± 2.3 and +16.4 ± 1.5 mV and (6.9 ± 0.6) × 10^11^ and (6.1 ± 1.0) × 10^11^ particles mL^−1^. RGD complexation was 7.73 × 10^4^ molecules per nanocapsule and Dox loading were 1.51 × 10^4^ and 7.64 × 10^4^ molecules per nanocapsule, respectively. RGD-functionalized nanocapsules had an improved uptake capacity by U87MG cells. Pareto chart showed that the cell viability was mainly affected by the Dox concentration and the period of treatment in both MCF-7 and U87MG. The influence of RGD-functionalization on cell viability was a determinant factor exclusively to U87MG.

## 1. Introduction

Cancer is a major public health problem worldwide. In the past decades, several nanoparticle-based drug delivery systems have obtained a great attention as promising approaches for tumor treatments [1,2,3,4]. The use of nanotechnology in drug delivery systems opened new opportunities to overcome limitations in therapeutics, such as low efficacy and high systemic toxicity of many drugs in clinical practice for cancer treatments [5].

Doxorubicin (Dox), an anthracycline antibiotic and anticancer drug, is used as a cytostatic agent in cancer chemotherapy [6]. Unfortunately, Dox clinical use is limited by dose-related cardiomyopathy, which becomes more prevalent with increasing cumulative doses [7]. Therefore, poor penetration of antitumor drugs into the extravascular tumor tissue is often a major factor limiting the efficacy of treatments [8,9].

To demonstrate efficacy, most of the anti-cancer drugs have to reach and internalize the cancer cells in a correct and appropriate concentration for an effective activity level [10]. Moreover, ligand-mediated chemotherapeutic drug targeting has emerged as a novel paradigm in targeting cancer contributing to selectively destroy cancer cells and reduce adverse effects to normal cells. Antecedently, various biocompatible nanoparticles with different structures and compositions have been identified as promising devices for drug targeting in the treatment of tumoral cells [2,11,12]. Surface functionalization of nanoparticles has traditionally been achieved using targeting groups, such as peptides or antibodies for specificity [10]. Conjugation of peptides at the surface of nanoparticles are in general developed using two approaches: by covalent binding of the protein to the surface of nanoparticle or by noncovalent interactions between the particle and protein [13].

Angiogenesis, the development of new blood vessels, plays a critical role in controlling tumor growth and metastasis. Several cell surface-proteins, over-expressed by tumor endothelial cells, contribute to cell invasion and angiogenesis. Among them, the role of α_v_β_3_ integrin in angiogenesis and tumor cell proliferation is well documented [14,15]. The α_v_β_3_ integrin is highly expressed (in different levels) in new blood vessels of tumors, as well as in cells of different solid tumors, such as glioblastomas, melanomas, ovarian cancer, breast cancer and prostate cancers [3,16,17,18]. The recognition of the widely expressed peptide sequence arginylglycylaspartic acid (RGD) favor its use as active targeting ligand of drugs [9,19,20,21]. Polymeric nanoparticles functionalized with RGD and containing DOX have been designed and developed for targeting tumor cells [22,23,24]. Using the phase inversion method and a conjugation with 1,2-distearoyl-sn-glycero-3-phosphoethanolamine-*N*-[maleimide(polyethylene glycol)-2000]. Hirsjärvi and co-workers [25] developed lipid nanocapsules grafted with a RGD cyclic-peptide (cRGD). Those nanocapsules are prepared with no hydrophobic polymer wall having their surface decorated with cRGD by a covalent bond with a lipophilic constituent material of the nanocapsules. The nanocapsules bound efficiently to cell integrins (U87MG cells) with effective internalization.

In our research group, we developed a kind of polymeric nanocapsules named lipid-core nanocapsules (LNC), which structure is an organogel as core (sorbitan monostearate and capric-caprylic triglyceride), a polymer wall (composed of a hydrophobic polyester) and a hydrophilic corona of polysorbate 80 micelles [26]. The lipid-core nanocapsules (LNC) showed their promising application in the treatment of cancer based on a variety of studies that we conducted with different encapsulated drugs carried and deliver to cells, tissues and organs [27,28,29,30]. Recently, we developed a liquid formulation containing Dox-loaded lipid-core nanocapsules (Dox-LNC) showing its in vitro antiproliferative activity against human breast cancer cell line (MCF-7 cells), which efficacy is related to the mechanism of cell uptake, mainly endocytosis mediated by caveolin [31]. In parallel, we developed an innovative strategy to decorate the surface of the lipid-core nanocapsules by coating them with lecithin-chitosan and coordinating ligands at the surface by forming an organometallic complex using zinc-II or iron-II [32]. The new supramolecular structures were complexed by a quick and versatile non-covalent bound with different molecules, such as an antibody fragment anti-LDL(-), scFv-anti-LDL(-) [32,33] and enzymes, such as laronidase [34] as well as bromelain [35]. Taking into account the above mentioned, we hypothesis that our previous liquid formulation containing Dox-LNC could be surface functionalized using the chitosan-metal ion-ligand-complex interfacial reaction to improve the tumor targeting by using RGD as active targeting ligand. Then, RGD-surface-functionalized-Dox-loaded lipid-core-nanocapsules were developed and physico-chemically characterized by diverse techniques and biologically evaluated using two different tumoral cell cultures (human breast adenocarcinoma cell line, MCF-7; and human glioblastoma astrocytoma, U87MG), which cell membranes express α_v_β_3_ integrin in different levels.

## 2. Results

### 2.1. Surface-Functionalized Metal-Complex Multi-Wall Nanocapsules

The lipid-core nanocapsules were prepared by self-assembling methodology injecting an organic phase into an aqueous phase following evaporation to remove acetone and concentrate the formulation. The nanocapsules were, then, coated with chitosan. The chitosan-coated nanocapsule surface was reacted with zinc acetate and RGD. RGD-MCMN was obtained as macroscopically homogenous white-opalescent liquid. Photon correlation spectroscopy (PCS) analysis showed hydrodynamic mean diameter (z-average) of 162 ± 6 nm, polydispersity index (PDI) of 0.11 ± 0.04 and zeta potential of +13.2 ± 1.9 mV. By NTA, the hydrodynamic mean diameter and particle number density (PND) were 192 ± 10 nm and (6.2 ± 1.1) × 10^11^ particles mL^−1^, respectively. Formulation was prepared with an RGD concentration of 843.64 μmol L^−1^. After ultrafiltration-centrifugation, we determined the yield of RGD complexation on the surface of the nanocapsules as 94.3 ± 5.9%. In this way, the interfacial reaction yielded 7.73 × 10^4^ RGD molecules per nanocapsule.

**Figure 1:**
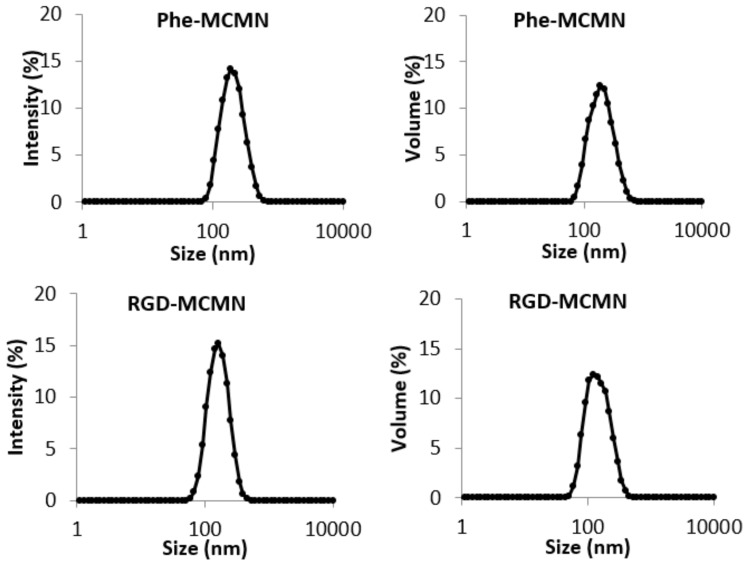
Diameter distribution profiles by intensity and by volume of particles determined by photon correlation spectroscopy (PCS) for surface-functionalized metal-complex multi-wall nanocapsules using phenylalanine or RGD as ligands (Phe-MCMN and RGD-MCMN).

In order to establish the influence of the RGD functionalization on the cell viability study, we prepared a formulation functionalized with phenylalanine (Phe), named here Phe-MCMN, as previously proposed as a control formulation in biological evaluations [33]. The new batch of this formulation had physico-chemical characteristics such as, z-average diameter of 188 ± 3 nm, PDI of 0.12 ± 0.02 and zeta potential of + 25 ± 2 mV. Phe-MCMN and RGD-MCMN showed unimodal distributions of diameters by PCS (Figure 1). It is noteworthy to mention the profiles expressed by intensity and by volume, in each case, are almost superimposed denoting the narrow size distributions of nanocapsules.

To determine the cytotoxicity of the nanoformulations, we prepared drug-loaded formulations containing Dox at 0.1 and 0.5 mg·mL^−1^. Therefore, the Dox-loaded nanoformulations functionalized with RGD were obtained as macroscopically homogeneous red-orange opalescent liquids. PCS and NTA showed nanoscopic populations, with mean diameters lower than 215 nm (Table 1). We also prepared Dox-loaded formulations functionalized with phenylalanine (Phe-MCMN (Dox100) and Phe-MCMN (Dox500)) to evaluate the influence of the chemical nature of the ligand on the cell viability. These nanoformulations were obtained as macroscopically homogeneous red-orange opalescent liquids. PCS and NTA analysis showed diameter profiles with narrow size distributions with mean diameters lower than 208 nm (Table 1). All nanoformulations containing Dox showed PDI lower than 0.10, PND close to 7 × 10^11^ nanocapsules per mL and zeta potential around +15 mV (Table 1).

Drug contents of 92.49 ± 7.17 and 457.55 ± 5.41 μg·mL^−1^ were experimentally determined for the Dox-loaded formulations prepared at 100 and 500 μg·mL^−1^, respectively. After ultrafiltration-centrifugation, drug encapsulation efficiencies were 91.58 ± 0.70% for RGD-MCMN (Dox100) and 92.61 ± 3.00% for RGD-MCMN (Dox500).

### 2.2. Cellular Uptake Studies

**Figure 2:**
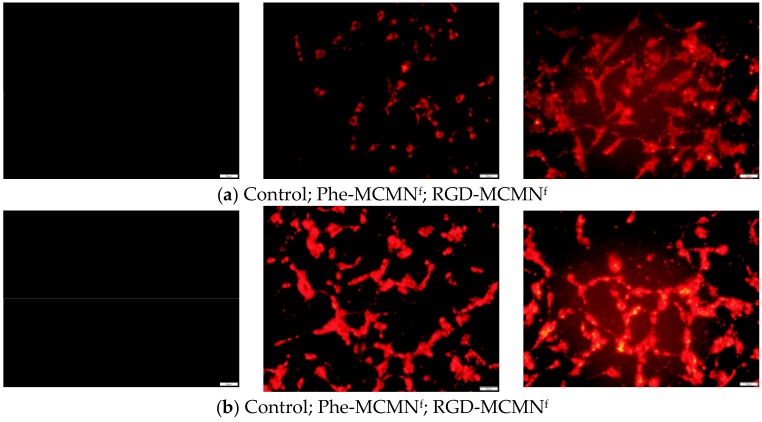
MCF-7 (**a**) and U87MG (**b**) cells were seeded at densities of 6.0 × 10^3^ and 20.0 × 10^3^ cells per well, respectively. Confocal microscopy analysis of cellular uptake after 24 h of treatment using the fluorescent-labeled nanoformulations (Phe-MCMN^f^ and RGD-MCMN^f^) at 5.15 × 10^−3^ μmol·L^−1^ of well). Photomicrographies were obtained at 20× magnification with an Olympus IX71 fluorescent microscope (Olympus Corporation, Tokyo, Japan) (bar = 100 μm).

We prepared fluorescent-labeled nanoformulations by using a blend of PCL with a PCL-Rhodamine B conjugate. After treating the MCF-7 and U87MG cells with the fluorescent labeled-nanoformulations, confocal microscopy images showed fluorescence emission for both Phe-MCMN^f^ and RGD-MCMN^f^ but in higher intensity for the latter in both cell cultures suggesting a higher RGD-MCMN^f^ cellular uptake (Figure 2).

**Figure 3:**
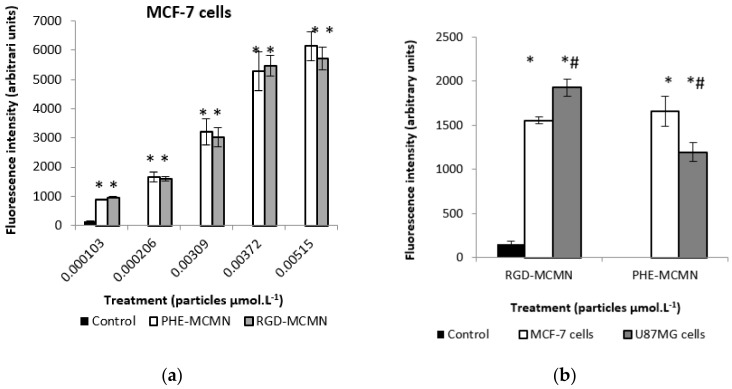
(**a**) Flow cytometry uptake profiles of Phe-MCMN^f^ or RGD-MCMN^f^ by MCF-7 cells treated for 24 h with 1.03 × 10^−4^, 2.06 × 10^−4^, 3.09 × 10^−3^, 3.72 × 10^−3^ and 5.15 × 10^−3^ μmol of nanocapsules per liter of well; and (**b**) uptake of RGD-MCMN^f^ and Phe-MCMN^f^ by MCF-7 and U87MG cells treated for 24 h with 1.03 × 10^−4^ μmol of particles per liter of well; control groups did not receive any treatment. Notes: Data are expressed as mean ± standard error. * Indicates significant differences from control; ^#^ indicates significant differences compared to the respective concentrations in (**a**,**b**); *^#^ indicates significant differences between cell lines. Differences were considered significant at *p* < 0.05.

On the other hand, by flow cytometry, despite the efficient uptake of Phe-MCMN and RGD-MCMN by human breast cancer cells MCF-7 cells, no statistical difference between the formulations was observed, whatever the nanocapsule concentration applied (Figure 3a). Therefore, we selected the lowest concentration of nanocapsules to treat the U87MG cells and to compare the cellular uptake of these formulations (Figure 3b). In this case, RGD-MCMN demonstrated an improved uptake capacity for U87MG cells, while Phe-MCMN presented higher uptake for MCF-7 cells.

### 2.3. Cell Viability Studies

**Figure 4:**
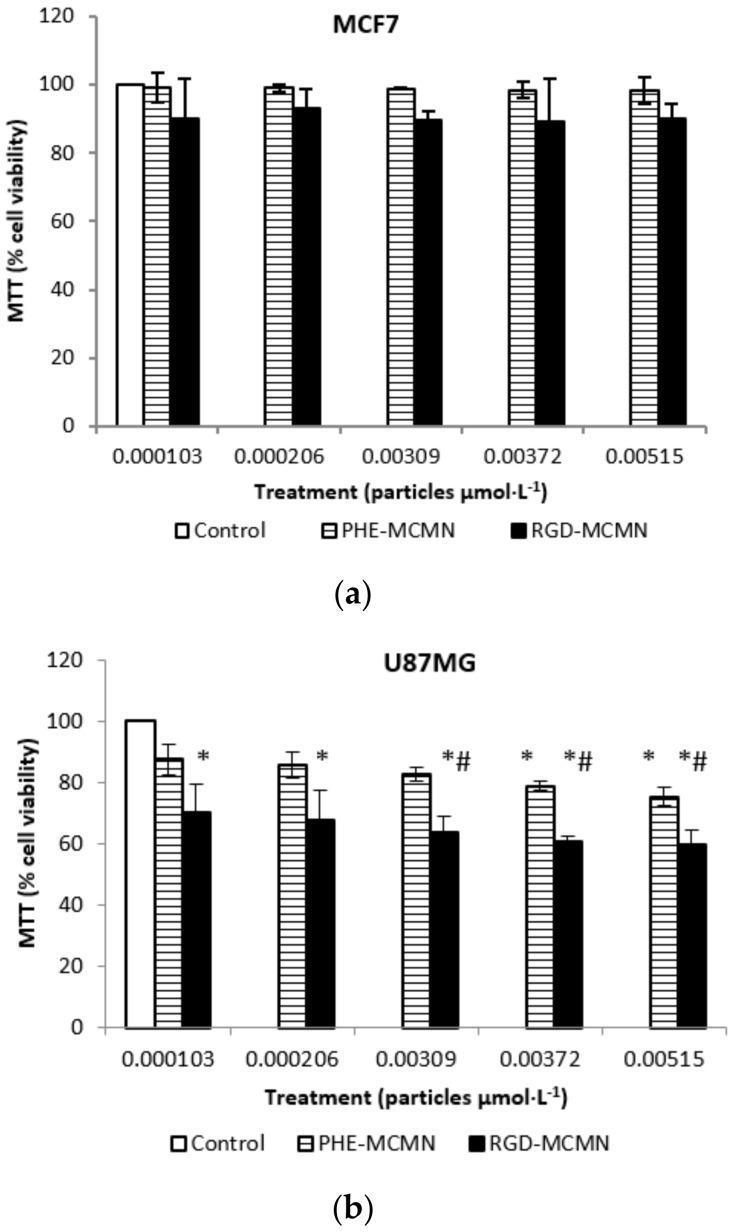
(**a**) Effect of Phe-MCMN and RGD-MCMN on the cell viability of human breast cancer cells (MCF-7 cell line) and (**b**) glioblastoma cells (U87MG cell line) using MTT assay. Cells were treated for 24 h with different nanocapsule concentrations (1.03 × 10^−4^, 2.06 × 10^−4^, 3.09 × 10^−3^, 3.72 × 10^−3^ and 5.15 × 10^−3^ μmol of particles per liter of well). Control group did not receive any treatment (100% cell viability). Notes: data are expressed as mean ± standard error. * Indicates significant differences compared to the control; ^#^ indicates significant differences compared to the respective concentrations; *^#^ indicates significant differences between cell lines. Differences were considered significant at *p *< 0.05.

The MTT assay was used to evaluate whether the drug-unloaded nanocapsule formulations (Phe-MCMN and RGD-MCMN) affect the cell viability of MCF-7 and U87MG cells after 24 h of treatment. To MCF-7 culture, no significant difference was observed in cell viability after treatments with increasing concentrations of Phe-MCMN and RGD-MCMN compared to the control group (Figure 4). While, to U87MG culture, all concentrations of RGD-MCMN applied showed significant decrease in cell viability compared to the control but only the highest concentrations of Phe-MCMN showed significant difference (Figure 4).

To determine the cytotoxicity of Dox-loaded nanoformulations, we carried out the MTT assay applying the formulations at concentrations of 1.03 × 10^−4^ and 2.06 × 10^−4^ μmol of nanocapsules per liter of well incubating them for 24 and 72 h. Dox-loaded nanocapsules were compared to Dox solution and the cell viability of the treated groups were calculated in relation to the control group (without treatment), which represents 100% of cell viability. During MTT assay, treated groups did not show significant alterations in the proliferation of the cell line when compared with the untreated group (control). After 24 and 72 h of treatment, all Dox treatments led to a significant decrease in cell viability in comparison with their respective control groups (*p *≤ 0.05).

MCF-7 cells were treated with drug concentration from 1.7 to 17 μmol of Dox per liter of well using two different concentrations of nanocapsules: 1.03 × 10^−4^ or 2.06 × 10^−4^ μmol of nanocapsules per liter of well. In the treatments with Doxorubicin concentration of 1.7 and 3.4 μmol·L^−1^, we used RGD-MCMN (Dox100), Phe-MCMN (Dox100) and Dox100, while for Doxorubicin concentration of 8.5 and 17 μmol·L^−1^ we used RGD-MCMN (Dox500), Phe-MCMN (Dox500) and Dox500. Taking into account the encapsulation efficiencies, the number Dox molecules per nanocapsule is about 1.51 × 10^4^ or 7.64 × 10^4^, respectively, for the formulations prepared using 100 and 500 μg of Dox per milliliter.

**Figure 5:**
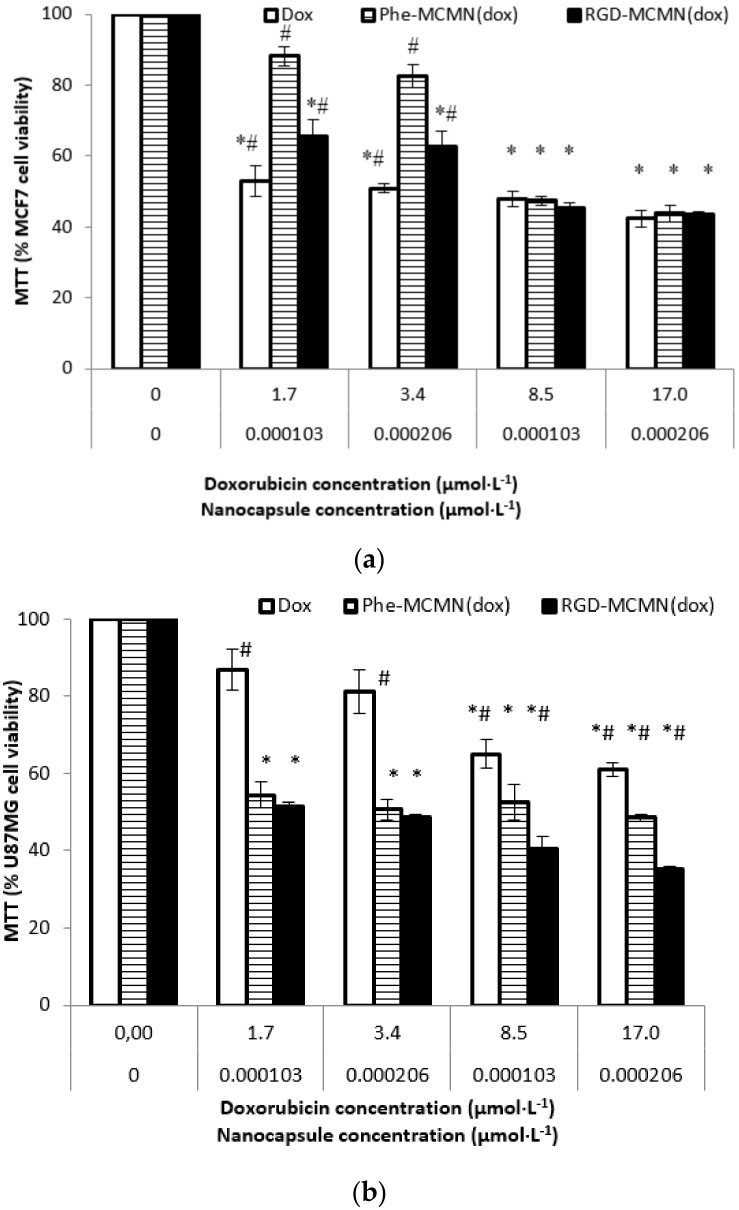
Cell viability by MTT assay after 24 h of treatment on (**a**) human breast cancer cells (MCF-7 cell line) and (**b**) glioblastoma cells (U87MG cell line) using nanocapsule concentrations at 1.03 × 10^−4^ and 2.06 × 10^−4^ μmol per liter of well and Doxorubicin concentrations at 1.7, 3.4, 8.5 and 17.0 μmol per liter of well. Control group did not receive any treatment (100% cell viability).Notes: Data are expressed as mean ± standard error. * indicates significant differences from control; ^#^ indicates significant differences compared to the respective concentrations; *^#^ indicates significant differences between cell lines. Differences were considered significant at *p *< 0.05.

After 24 h of incubation, MTT assay (Figure 5) showed a cell viability from 65.67 ± 4.73% to 43.59 ± 0.81% for RGD-MCMN (Dox), 88.19 ± 2.70% to 43.72 ± 2.26% Phe-MCMN (Dox) and 52.93 ± 4.41% to 42.35 ± 2.40% for Dox. The treatments with 1.7 and 3.4 μmol of Doxorubicin per liter of Phe-MCMN (Dox) formulation did not present significant difference (*p* > 0.05) compared to the control (100% of viability). Furthermore, no statistical difference was determined comparing the formulations containing the highest concentration of Dox (*p* > 0.05). Nevertheless, regarding treatments with 1.7 and 3.4 μmol of Dox per liter of well using RGD-MCMN (Dox), a greater decrease in the viability of MCF-7 cells was observed compared to Phe-MCMN (Dox) (*p* < 0.05). MTT assay carried out with U87MG cells, after 24 h of treatment with similar Dox concentrations using the same formulations showed a cell viability from 51.45 ± 0.96% to 35.24 ± 0.51% for RGD-MCMN (Dox), from 54.43 ± 3.26% to 48.48 ± 0.94% for Phe-MCMN (Dox) and from 86.87 ± 5.24% to 61.10 ± 1.70% for Dox. The treatments using 1.7 and 3.4 μmol of Dox per liter of well did not show significant difference (*p* > 0.05) compared to the control (100% of viability). No statistical difference was determined comparing RGD-MCMN (Dox) and Phe-MCMN (Dox) using 1.7 and 3.4 μmol of Dox per liter of well (*p* > 0.05). In addition, nanocapsule formulations showed higher cytotoxicity than Dox in all concentrations of treatment (*p* < 0.05) and RGD-MCMN (Dox) showed the highest decrease of viability among the formulations.

**Figure 6:**
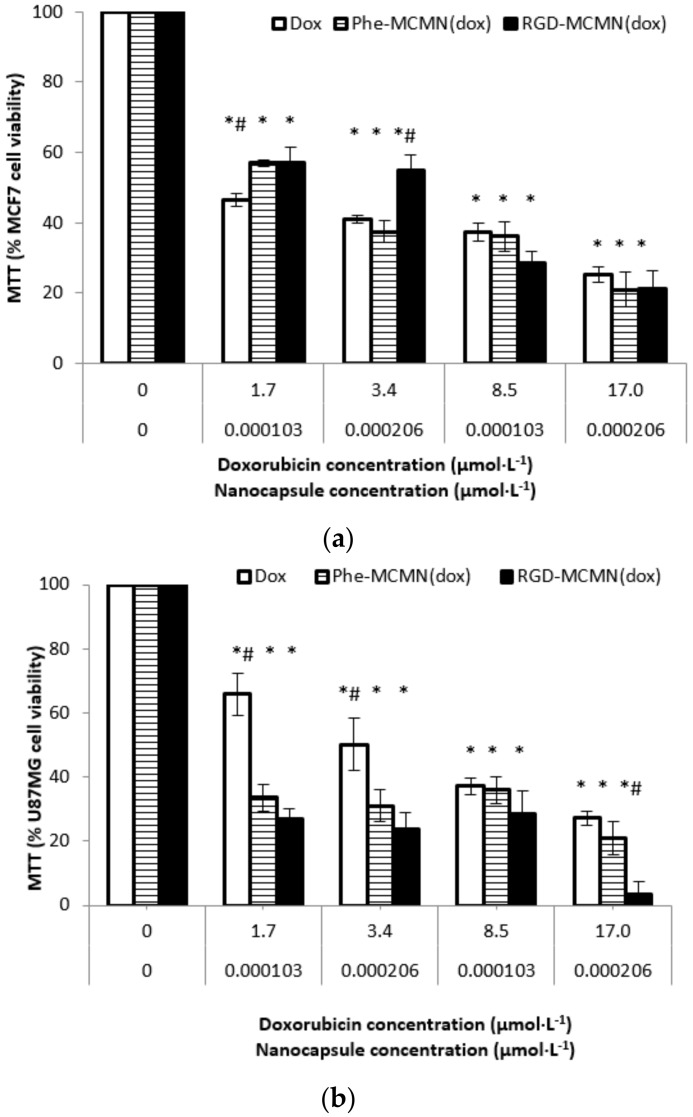
Cell viability by MTT assay after 72 h of treatment on (**a**) human breast cancer cells (MCF-7 cell line) and (**b**) glioblastoma cells (U87MG cell line) using nanocapsule concentrations at 1.03 × 10^−4^ and 2.06 × 10^−4^ μmol per liter of well and Doxorubicin concentrations at 1.7, 3.4, 8.5 and 17 μmol·per liter of well. Control group did not receive any treatment (100% cell viability). Notes: data are expressed as mean ± standard error. * Indicates significant differences from control; ^#^ indicates significant differences compared to the respective concentrations; *^#^ indicates significant differences between cell lines. Differences were considered significant at *p *< 0.05.

After 72 h of incubation, MTT assay (Figure 6) in MCF-7 showed cell viability from 57.17 ± 4.20% to 21.15 ± 5.00% for RGD-MCMN (Dox), from 57.04 ± 0.87% to 20.93 ± 5.00% for Phe-MCMN (Dox) and from 46.45 ± 1.77% to 25.26 ± 2.16% for Dox. All treatments presented significant difference (*p* < 0.05) compared to the control. Only RGD-MCMN (Dox) demonstrated significant difference (*p* < 0.05) compared to other treatments in similar concentration. After 72 h, MTT assay carried out with U87MG cells, showed cell viability from 26.77 ± 3.21% to 3.21 ± 4.00% for RGD-MCMN (Dox), from 33.62 ± 4.17% to 20.93 ± 5.00% for Phe-MCMN (Dox) and from 65.89 ± 6.56% to 27.26 ± 2.16% for Dox. All treatments presented significant difference (*p* < 0.05) compared to the control. Dox presented significant difference (*p* < 0.05) compared to the treatments using 1.7 and 3.4 μmol of Dox per liter of well. RGD-MCMN (Dox) showed the highest decrease in cell viability compared to the other treatments using 17 μmol of Dox per liter of well (*p* < 0.05).

**Figure 7:**
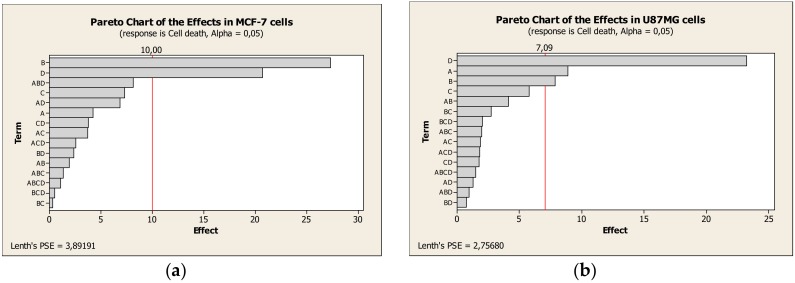
Standardized effects of A = functionalization, B = Dox concentration, C = nanocapsule concentration, D = period of incubation and their interaction effects on the cell death ((**a**) MCF-7 cells; and (**b**) U87MG cells).

To analyze the results considering all parameters (factors) varying among the groups of treatments, a Pareto chart was constructed (Figure 7). The factors that most influenced the decrease in cell viability or the interaction of factors were evaluated. The vertical line indicates the minimum value of effect for the significant cell death; and the greater the effect, the greater the significance. In Pareto chart for MCF-7 cells, Dox concentration and period of treatment were the factors that most influenced the results. While, for U87MG cells, the period of treatment, the surface-functionalization and the Dox concentration were the factors that most influenced the results of cell viability.

## 3. Discussion

In spite of much ongoing research about cancer, the treatment of tumors still proves difficult. Therefore, various nanoparticles based drug delivery systems have been studied [2,27,29]. The targeting of RGD-modified nanoparticles to tumor vasculature is a promising strategy for tumor-targeting treatment, because some tumor cells within solid tumors would overexpress α_ν_β_3_ integrin, which can specifically recognize the peptide RGD [20,23,36,37,38]. Buckley and co-workers [39] described that RGD-containing peptides enter cells and directly induce autoprocessing and enzymatic activity of procaspase-3, a pro-apoptotic protein.

We developed the RGD-surface-functionalized metal-complex multi-wall nanocapsules by adapting the methodology we previously described [32] to obtain scFv-anti-LDL(-)-surface-functionalized multi-wall-nanocapsules. Different formulations were prepared to compare the effect of the encapsulated drug (Dox) and the ligand (RGD) on the cell viability of MCF-7 and U87MG. To evaluate the influence of the integrin recognition on the cell viability assays, we also prepared formulations without Dox but functionalized with Phe (Phe-MCMN) or RGD (RGD-MCMN). RGD-MCMN was more efficient to inhibit the growth of U87MG cells (human glioblastoma cells expressing high levels of integrin α_ν_β_3_) than similar concentrations of Phe-MCMN. In parallel, none of those formulations was cytotoxic to MCF-7 cells in similar experimental conditions.

Integrins are heterodimeric membrane glycoproteins composed of non-covalently associated α and β subunits. The αvβ3 integrin is highly expressed in U87MG cells, which express approximately 10^5^ αvβ3 receptors per cell [40]. However, according to Zhang et al. [41] the quantification of αvβ3 integrin expression in cells or tissues by immunoblotting is technically challenging because both anti-αv and anti-β3 antibodies are thus needed to confirm the presence of αvβ3 integrin. Liu and co-workers [38] could not detect β3 integrin in MCF-7 cells by either RT-PCR or western blots. Additionally, the absence of β3 integrin they showed the inability of MCF7 cells to form αvβ3 heterodimers. Besides, αvβ3 integrin is not expressed on mature vessels or on non-neoplastic epithelium [41].

The supramolecular structure of the multi-wall nanocapsules is based on secondary bonds among the lipids and the lipids and PCL, as well as on the electrostatic interactions between the ammonium groups of chitosan and the negatively charge groups present in Lipoid S75 (lecithin). The addition of zinc acetate solution leads to the chitosan-metal complex formation. This complex is highly reactive [32,42] and, then, capable of reacting with different molecular compounds, such as RGD. The formulations developed in this study presented unimodal and nanometric particle sizes, which characteristics are consistent with the diameters usually observed for polymeric nanocapsules dispersed in water prepared by solvent displacement method. A narrow particle distribution was observed for the formulations, indicated by the low polydispersity. The zeta potential values were positive as previously described for chitosan-coated nanocapsules [32].

To the Dox-loaded nanocapsules, the encapsulation efficiency (*EE*%) was higher than 90% for both drug concentrations (100 or 500 μg·mL^−1^), demonstrating the high capacity of Dox loading on these nanocapsules. The high *EE*% in the present study is attributed primarily to the neutral drug form interacting with the lipid-core of the nanocapsules and also to probable electrostatic interactions between amine groups of Dox molecules and the oxygenated groups of Lipoid S75 (lecithin). Similarly, as previously described, Dox can be associated with the nanoparticles by electrostatic interactions [43,44,45,46,47]. Considering the organometallic complex at the nanocapsule surface, the binding of RGD-peptide on the nanocapsule was 94.3 ± 5.9%. These results demonstrated adequate encapsulation efficiency and surface functionalization to conduct the in vitro biological evaluations. The cell viability study after 24 h of treatment with blank formulations (RGD-MCMN and Phe-MCMN) was conducted to evaluate their toxicity to the MCF-7 and U87MG cells. Besides that, our results also showed potent cytotoxicity of formulations containing Dox when functionalized with RGD. After 72 h of treatment, these formulations were able to eliminate almost 100% of the human glioblastoma cells. Moreover, they also presented high cytotoxicity effect to MCF-7 cells. Previously, Yu and co-workers [40] demonstrated that Dox-loaded nanoparticles functionalized with RGD showed much higher effect to U87MG cells, than to MCF-7 cells, which presents low expression of this integrin. Recently, magnetic RGD cycle-peptide-functionalized nanoparticles containing Dox resulted in the low efficiency of targeted drug delivery in MCF7 cells and were able to dramatically increase the drug efficacy to U87MG cells probably due to the receptor-mediated endocytosis. Moreover, the applied magnetic fields further affected the cytotoxicity of magnetic nanoparticles because the concentrations of drug nanocarriers around cancer cells can be manipulated by remote control using small magnets for these cells [48]. In addition, Dox-encapsulated nanoparticles formed by poly(RGD-*co*-β-amino ester) and Dox-polymeric nanoparticles functionalized with RGD had higher cytotoxicity than nanoparticles prepared without RGD in U87MG cells [24]. Besides these, in recent years, other authors have described the improvement in transport of Dox into tumor cells with targeting of RGD-nanoparticles using different materials and methods of preparation [37,49,50,51,52,53].

Different from the nanoparticles cited in these studies, we used surface coordination ligands for non-covalent binding to RGD. In general, the nanoparticle surface functionalization with ligands, such as peptides is carried out primarily by conjugation methods. Conjugation of ligands can be random or site specific and random conjugation can cause a lack of the ligand specificity. Thus, the ability to specifically bind to its target receptor might be decreased. Therefore, a strategy of surface coordination ligands for non-covalent binding can ensure full ligands activity [32].

Phe-MCMN (Dox) showed promising results regarding the cytotoxicity in MCF-7 and U87MG cells. Those findings are likely explained by the aromatic residue of Phe as lateral chain, which can cause an increase in rupture capacity of the endosomal membrane as previously reported by Plank [54]. Furthermore, hydrophobic microparticles and dendrimers containing Phe have led to the destabilization or rupture of membrane in dendritic cells and macrophages [55,56]. Other studies also describe higher activity for membrane rupture by polymers containing Phe and its use to deliver intracellularly drugs [57,58]. In parallel, Ho and co-workers [59] developed pH-responsive biomimetic pseudo-peptides synthesized by grafting L-phenylalanine onto the pendant carboxylic acids of a polyamide, poly(L-lysine isophthalamide) polymers containing Phe and showed effective penetration into tumor models (HeLa—cervical cancer cells), leading to improvements in therapeutic delivery for extracellular and intracellular tumors.

Pareto chart allowed confirming the importance of surface functionalization with RGD to decrease U87MG cell viability. In addition to this effect, Dox concentration and period of treatment were also significant to decrease cell viability for both tumor cells. Our hypothesis was validated. The significant effect of functionalization to inhibit the growth of U87MG cells is a consequence of the RGD targeting effect on these cells, which have specific integrin to bind RGD [14,24,48,50].

The fluorescence from nanocapsules was observed to U87MG and MCF-7 cells, showing the uptake of RGD-MCMN^f^ and Phe-MCMN^f^ by both tumor cells. Therefore, the cellular uptake study showed that the increase in the nanocapsule concentration enhanced the fluorescence detected indicating a correlation to the cellular uptake. Besides, the similarity of Phe-MCMN and RGD-MCMN to MCF-7 cells viability, as well as the highest cellular uptake of RGD-MCMN^f^ by U87MG cells corroborate to the Pareto chart analysis, which demonstrated the importance of the RGD functionalization to decrease the viability of U87MG cells. Wang and co-workers [22] carried out cellular uptake studies with B16F10, DU145, MD-MB231 and MCF-7 cells, which have different expression levels of α_v_β_3_ as well as RGD-nanoparticles and showed higher cellular uptake to B16F10 and MD-MB231 cells, with greater expression of α_v_β_3_ integrin on the membrane.

We previously investigated the toxicity of drug-unloaded LNC formulation prepared with PCL using in vivo models after oral and intraperitoneal administrations, respectively, to Caenorhabditis elegans [60] and Wistars rats [61]. The results showed that LNC is a safe formulation. Besides that, phenylalanine-surface functionalized nanocapsules (Phe-MCMN), which organometallic complex was also prepared with Zn^2+^, caused no significant changes in the cell death (apoptosis + necrosis) in both the RAW 264.7 macrophages and HUVEC cell lines [33]. Even though RGD-surface-functionalized metal-complex multi-wall nanocapsules can be considered a supramolecular derivative of LNC and Phe-MCMN, further studies are necessary to access their safety.

## 4. Materials and Methods

### 4.1. Materials

Doxorubicin hydrochloride (98–102%), Span^®^ 60 (sorbitan monostearate), Poly(ε-caprolactone) (α,ω-dihydroxy functional polymer, M_n_ 10 kg·mol^−1^, M_w_ 14 kg·mol^−1^), chitosan low molar weight (M_w_ 50–190 kg·mol^−1^, 75–85% deacetylated polymer), zinc acetate (catalog number 383317), QuantiPro^™^ BCA Assay Kit, 3-(4,5-dimethylthiazol-2-yl)-2,5-diphenyltetrazolium bromide (MMT) and arginylglycylaspartic acid (RGD) were obtained from Sigma-Aldrich (St. Louis, MO, USA). Caprylic/capric triglyceride and Tween^®^ 80 (polysorbate 80) were delivered by Delaware (Porto Alegre, Brazil). Lipoid^®^ S75 (soybean lecithin) was obtained from Lipoid (Ludwigshafen am Rhein, Germany). Solvents used were of analytical or pharmaceutical grades. For cell culture studies, the human breast cancer cell line (MCF-7) and the human glioblastoma cell line (U87 MG) were obtained from the American Type Culture Collection (Rockville, MD, USA; ATCC HTB-22 and HTB-14). Dulbecco’s modified Eagle’s medium (DMEM low) and fetal bovine serum (FBS) were purchased from Invitrogen (Waltham, MA, USA).

### 4.2. Preparation of Surface-Functionalized Metal-Complex Multi-Wall Nanocapsules (MCMN)

#### 4.2.1. Neutralization of Doxorubicin Hydrochloride

Dox (base) was obtained as previously reported [62]. Briefly, Dox·HCl was dissolved in MilliQ^®^ water at 1 mg·mL^−1^. The orange-red solution was transferred to a separating funnel, added of trimethylamine (turning to violet) and extracted with chloroform (orange). The process was repeated until the aqueous phase was colorless. The organic phases were combined, dried under anhydrous sodium sulfate, filtered and evaporated under reduced pressure (Büchi, Flawil, Switzerland). Batches of 0.001 or 0.005 g were produced. The Dox (base) was dissolved in acetone and the solution was used to produce the formulations as described below.

#### 4.2.2. Preparation of Lecithin-Polysorbate 80-Coated Lipid-Core Nanocapsules (LNC)

Poly(ε-caprolactone) (0.100 g), Span^®^ 60 (0.040 g) and caprylic/capric triglyceride (0.120 g) were dissolved in acetone (25 mL) containing Dox (base) at 40 °C. Then, a solution of Lipoid S75^®^ (0.090 g) in ethanol (7 mL) was added into the organic phase. This organic solution was injected into an aqueous phase (53 mL) containing Tween^®^ 80 (0.080 g) under magnetic stirring at 40 °C. After 10 min, the organic solvents were evaporated and the formulation concentrated under reduced pressure at 40 °C using a rotary evaporator (Büchi, Flawil, Switzerland). A formulation containing no drug was also prepared as described above using acetone without Dox.

#### 4.2.3. Chitosan Coating

Chitosan coating was performed as described previously [32,33]. A 7 mg·mL^−1^ chitosan solution was prepared by dispersing chitosan in 1% acetic acid aqueous solution (MilliQ^®^ water). The solution was filtered (0.45 μm, Millipore, Burlington, MA, USA) and dropwise added (1 mL) into lecithin-polysorbate 80-coated lipid-core nanocapsule aqueous dispersion (9 mL) under magnetic stirring (500 rpm). The reaction was carried out under magnetic stirring for 3 h at room temperature (20 °C).

#### 4.2.4. Surface Functionalization

The surface functionalization was carried out by forming an organometalic complex adapting the methodology previously described for nanocapsules decorated with an antibody fragment [32]. A 1 mg·mL^−1^ zinc acetate aqueous solution (MilliQ^®^ water) was prepared at 25 °C. Then, 250 μL of this solution was added into 9.75 mL of chitosan-lecithin-polysorbate 80-coated lipid-core nanocapsule aqueous dispersion under magnetic stirring (500 rpm). After 1 min, 351.21 μL of this complexed nanocapsule aqueous dispersion was added of 648.79 μL of a 450 μg·mL^−1^ RGD aqueous solution under magnetic stirring (500 rpm) at room temperature (20 °C).

In parallel, to obtain phenylalanine-functionalized nanocapsules, 250 μL of the zinc acetate solution was added into 9.75 mL of chitosan-lecithin-polysorbate 80-coated lipid-core nanocapsule aqueous dispersion under magnetic stirring (500 rpm). After 1 min, the reaction medium (10 mL) was added of 250 μL of 7.6 mg·mL^−1^ phenylalanine aqueous solution under magnetic stirring (500 rpm), using a ligand/metal molar proportion of 3:1.

Formulations prepared without Dox were named RGD-MCMN and Phe-MCMN, while formulations containing Dox at 100 and 500 μg·mL^−1^ were respectively named RGD-MCMN (Dox100), RGD-MCMN (Dox500), Phe-MCMN (Dox100) and Phe-MCMN (Dox500).

For the cell uptake study, fluorescent-labeled formulations were prepared using a PCL derivative as previously described by Poletto and co-workers [63]. The conjugate was obtained by the esterification of Poly(ε-caprolactone) (PCL) with rhodamine B (PCL-RhoB) using an acid activating agent (carbodiimide). In this case, a blend of PCL (0.090 g) and PCL-RhoB (0.010 g) was used to constitute the polyester wall of the nanocapsules using the methodology described above.

### 4.3. Photon Correlation Spectroscopy and Laser Doppler Micro-Electrophoresis

Photon correlation spectroscopy (PCS) and laser Doppler micro-electrophoresis were carried out in a Zetasizer Nanoseries^®^ ZS instrument (Malvern, UK). Mean diameter by intensity (z-average) was determined by PCS using the Cumulants method. The samples (20 μL) were diluted to 10 mL in purified water (1:500 v/v) and zeta potential was determined by laser Doppler micro-electrophoresis by Zetasizer Nanoseries^®^ ZS instrument (Malvern, UK), after dilution of the samples in 10 mmol·L^−1^ NaCl aqueous solution (1:500 v/v).

### 4.4. Nanoparticle Tracking Analysis

The particle number density (particles mL^−1^), hydrodynamic diameter and median diameter by number of particles were determined by nanoparticle tracking analysis (NTA) in a NanoSight LM10 instrument (NanoSight Ltd., Salisbury, UK) using the provided analytical software NTA 2.0 (NanoSight Ltd., Salisbury, UK). This analysis allowed the observation of the Brownian motion recording the light scattered by the individual nanoparticles. Samples were diluted 5000× in ultrapure water (Milli-Q^®^; Merck KGaA, Darmstadt, Germany) and injected into the sample chamber. Each video clip was captured over 60 s.

### 4.5. Drug Content and Encapsulation Efficiency

Dox was quantified by liquid chromatography (HPLC), adapting a method previously described [64]. The system was a HPLC Shimadzu equipped with a CBM-20A controller, a SPD-M20AV detector, a degasser DGU-20A5, a LC-20AT pump and a SIL-20A auto-sampler (Kyoto, Japan). A guard column Cartridges C18 (4.0 × 3.0 mm, Phenomenex, Torrance, CA, USA) and a column RP-18 (150 mm × 4.6 mm × 5 μm ODS2 Waters Spherisorb^®^, Waters Corporation, Milford, MA, USA) were used as stationary phase and a solution of 0.1% trifluoroacetic acid and acetonitrile (50:50, v/v) at apparent pH 2.65, as the mobile phase. The flow rate was 1.0 mL·min^−1^, using an injection volume of 50 μL and detection at 254 nm.

To determine the experimental drug content (total concentration of Dox in the formulation), Dox was extracted from the formulations (0.5 mL) using acetonitrile (5 mL). The mixtures were stirred for 5 min and filtered (0.45 μm; Merck KGaA) for injection (HPLC). To determine the encapsulation efficiency (*EE*%), the non-encapsulated fraction of DOX was also determined. The ultrafiltration-centrifugation technique was carried out using 10 kDa cutoff units (Merck KGaA) and centrifugation at 1844× *g* for 5 min (Sigma^®^ 1-14; SIGMA Laborzentrifugen GmbH, Osterode am Harz, Germany). The non-encapsulated fraction of Dox (fraction of drug dissolved in the continuous phase) was determined by HPLC in the ultrafiltrate without dilution. Then, *EE*% was calculated using Equation (1).

*EE*% = (*C_t_*− *C*_non-encap_)/*C_t_* × 100 (1)

where *C_t_* is the experimental drug content and *C*_non-encap_ is the fraction of Dox dissolved in the water phase.

### 4.6. Yield of RGD Complexation on the Nanocapsule Surface

The yield of RGD complexation on the nanocapsule surface was determined by an indirect method. The non-bound fraction of RGD in the formulations was quantified by a colorimetric method (QuantiPro™ BCA Assay Kit, Sigma-Aldrich, St. Louis, MO, USA). According to the manufacturer’s instructions, a calibration curve was prepared using dilutions of bovine serum albumin (BSA) from 3 to 27 μg·mL^−1^. The reaction product has absorbance at 562 nm recorded on a plate reader (Spectramax, Molecular Devices, Sunnyvale, CA, USA). To isolate the non-bound fraction of RGD, the RGD containing samples were placed in ultrafiltration-centrifugation units (30 kDa; Merck KGaA) centrifuged at 1840× *g* Relative Centrifugal Force (RCF) during 5 min (Sigma^®^ 1-14; SIGMA Laborzentrifugen GmbH, Germany). The ultrafiltrate was assay using the colorimetric method and the RGD concentration determined using the calibration curve. The yield of RGD complexation on the nanocapsule surface (Yield%) was determined using Equation (2).

Yield% = (*RGD_total_* − *RGD_non-bound_*)/*RGD_total_* × 100 (2)

where *RGD_total_* is the RGD concentration in the formulation and *RGD_non-bound_* is the RGD concentration dissolved in the ultrafiltrate.

### 4.7. Cell Culture Assays

Cell cultures were grown in DMEM (Dulbecco’s Modified Eagle) supplemented with 10% of fetal bovine serum (FBS), 0.1% amphotericin, 1% penicillin/streptomycin (Invitrogen, Waltham, MA, USA) in an atmosphere of 5% CO_2_/95% air at 37 °C. Fresh medium was replaced every other day.

### 4.8. Confocal Microscopy Analysis

For confocal microscopy analysis cellular uptake, MCF-7 and U87MG cells were seeded at densities of 6.0 × 10^3^ and 20.0 × 10^3^ cells per well, respectively, in 24-well plates. After reaching 60% to 70% of confluency, cells were treated with the fluorescent-labeled RGD-MCMN^f^ or fluorescent-labeled Phe-MCMN^f^ using 5.15 × 10^−3^ μmol of particles per liter of well. The volume per well was completed to 500 μL with DMEM. After incubation for 24 h at 37 °C, formulations were removed and cells were washed with PBS (0.5 mL). After the second addition of PBS (0.5 mL), photomicrographies were obtained at 20× magnification with an Olympus IX71 fluorescent microscope (Olympus Corporation, Tokyo, Japan) equipped with the CellSens Standard software (Olympus Corporation, Tokyo, Japan).

### 4.9. Quantitative Cellular Uptake Studies

Quantitative cellular uptake of fluorescent-labeled RGD-MCMN was evaluated by flow cytometry. MCF-7 and U87MG cells were seeded at densities of 6.0 × 10^3^ and 20.0 × 10^3^ cells per well, respectively, in 24-well plates. After reaching 60% to 70% of confluency, cells were treated with the fluorescent-labeled RGD-MCMN^f^ or fluorescent-labeled Phe-MCMN^f^ using the concentrations of nanocapsules ranged from 1.03 × 10^−4^ to 5.15 × 10^−3^ μmol of particles per liter of well. The volume per well was completed to 500 μL with DMEM. After incubation for 24 h at 37 °C, formulations were removed and cells were gently washed with PBS buffer (3 times), detached with trypsin-EDTA, harvested and analyzed in a flow cytometer BD FACSVerse System (BD, Franklin Lakes, NJ, USA) equipped with a PE filter in order to determine fluorescence intensity emitted by the fluorescent-labeled RGD-MCMN or fluorescent-labeled Phe-MCMN.

### 4.10. Cell Viability Studies

The 3-(4,5-dimethylthiazol-2-yl)-2,5-diphenyltetrazolium bromide (MTT) assay was used to estimate MCF-7 and U87MG cell viabilities. Cells were seeded 1500 cells per well for MCF-7 and 5000 cells per well for U87MG into 96-well plates. After 48 h, the medium (DMEM) was removed, the adherent cells were treated with each formulation and the volume completed to 100 μL with DMEM. The concentrations of nanocapsules in the treatments ranged from 1.03 × 10^−4^ to 5.15 × 10^−3^ μmol of particles per liter of well. The micromolar concentrations of nanocapsules were calculated using the Avogadro number (6.023 × 10^23^) considering the particle number density (PND) determined as described above using NTA. The cells were incubated for 24 and 72 h. After incubation, the medium was removed and each well was washed with phosphate-buffered saline to the addition of 0.5 mg·mL^−1^ MTT solution. The cells were incubated at 37 °C for 3 h. The MTT solution was removed and DMSO (100 μL) was added to dissolve the formazan crystals. Thus, the absorbance was measured at 560 nm and 630 nm on a microplate reader (Spectramax M2e and v 5.4.1, SoftMax Pro Software Interface; Molecular Devices, Sunnyvale, CA, USA). The results were converted to cell viability, expressed as the percentage of cell viability against the control group, which did not receive any treatment (100% of cell viability).

### 4.11. Statistical Analysis

All data are expressed as mean values ± standard deviation calculated from at least three independent experiments. Statistical analyses were performed using GraphPad Prism 5.0 (GraphPad Software Inc., La Jolla, CA, USA). The level of significance was set to *p* < 0.05. For the cell viability results, the software MINITAB version 16.0 (Minitab Inc., State College, PA, USA) was used to plot the Pareto chart. A graphical display of the effect by factor was given in a Pareto chart, which analyzes the magnitude and the importance of each variable effect. The length of the bars in the chart is proportional to the effect. A factor was considered as “statistically significant” if its standardized effect exceeded a threshold. A line in the Pareto chart indicated the threshold for a test at the *p*-value of 0.05.

## 5. Conclusions

RGD surface modified LNC were successfully developed. Therefore, different Dox concentrations were nanoencapsulated into these nanocapsules. The cellular uptake of RGD-MCMN was significantly enhanced to the integrin-overexpressed cell lines (U87MG) compared to the integrin-deficient cell lines (MCF-7). Moreover, Pareto chart showed that the cell viability was mainly affected by Dox concentration and period of treatment in both MCF-7 and U87MG. The influence of RGD-functionalization on cell viability was a determinant factor exclusively to U87MG. We believe that this study provides a facile strategy towards the development of active-targeting drug-loaded nanocarriers for tumor therapy via integrin mediation. In this way, in vivo pre-clinical trials can be performed to evaluate the innovative formulations as a new chemotherapeutic strategy for drug delivery to tumor cells without damaging normal cells using α_v_β_3_ integrin targeted antiangiogenic strategies for cancer treatment by intravenous administration.

## Figures and Tables

**Table 1:** Physicochemical characterization of formulations containing Doxorubicin by photon correlation spectroscopy (PCS), nanoparticle tracking analysis (NTA) and electrophoretic light scattering (ELS) (mean ± SD).

Formulation	PCS		NTA		ELS
	Z-Average Diameter (nm)	Mean Size (nm)	PDI	PND (×10^11^ Particles mL^−1^)	Zeta Potential (mV)
RGD-MCMN (Dox100)	146 ± 20	128 ± 12	0.09 ± 0.05	6.9 ± 0.6	+13.8 ± 2.3
RGD-MCMN (Dox500)	215 ± 25	135 ± 21	0.09 ± 0.03	6.1 ± 1.0	+16.4 ± 1.5
Phe-MCMN (Dox100)	186 ± 19	110 ± 25	0.08 ± 0.05	6.9 ± 0.3	+17.1 ± 0.7
Phe-MCMN (Dox500)	208 ± 22	132 ± 15	0.09 ± 0.02	6.5 ± 0.7	+16.5 ± 1.4
